# Half metal in two-dimensional hexagonal organometallic framework

**DOI:** 10.1186/1556-276X-9-690

**Published:** 2014-12-20

**Authors:** Hao Hu, Zhengfei Wang, Feng Liu

**Affiliations:** Frontier Institute of Science and Technology, Xi’an Jiaotong University, Xi’an, 710054 China; Department of Materials Science and Engineering, University of Utah, Salt Lake City, UT 84112 USA; Collaborative Innovation Center of Quantum Matter, Beijing, 100084 China

**Keywords:** Half metal, 2D hexagonal organometallic framework, Triphenyl-transition metal, Spintronics device

## Abstract

Two-dimensional (2D) hexagonal organometallic framework (HOMF) made of triphenyl-metal molecules bridged by metal atoms has been recently shown to exhibit exotic electronic properties, such as half-metallic and topological insulating states. Here, using first-principles calculations, we investigate systematically the structural, electronic, and magnetic properties of such HOMFs containing 3d transition metal (TM) series (Sc to Cu). Two types of structures are found for these HOMFs: a buckled structure for those made of TMs with less half-filled 3d band and a twisted structure otherwise. The HOMFs show both ferromagnetic and antiferromagnetic properties, as well as nonmagnetic properties, due to the electronic configuration of the TM atoms. The V, Mn, and Fe lattices are ferromagnetic half metals with a large band gap of more than 1.5 eV in the insulating spin channel, making them potential 2D materials for spintronics application.

## Background

Half metals, which act as conductor for electrons of one spin orientation, but as insulator for those of opposite spin orientation, have attracted much recent interest [1]. Due to their 100% spin polarization near the Fermi level, the half metals can provide purely spin-resolved electric current, holding great promise for future spintronics and nanoelectronic devices. Most known half metals are ferromagnets, with a few exceptions of half-metallic antiferromagnets [2]; on the other hand, most magnets are not half metals. Although some types of magnets have been theoretically predicted to be half metals, most of them do not have high enough spin polarization at room temperature for device applications [3]. As such, much effort has been devoted to searching for new half-metallic materials, including organic half metals and half-metallic nanostructures.

In the last decade, 2D materials made of a single atomic layer have drawn much interest from both fundamental and practical points of view [4]. Magnetic as well as half-metallic properties of 2D materials are especially attractive topics of study, because they can be tuned by manipulating geometry [5, 6], doping, and adsorption [7–17]. One way to achieve magnetic 2D materials with half metallicity is via self-assembled growth of organometallic frameworks using organometallic compounds. For example, the single layer organometallic framework of transition metal (TM)-phthalocynine has been synthesized [18], and first-principle calculations demonstrated its half metallicity [19]. Recently, the 2D lattice of hexagonal organometallic frameworks (HOMFs) [20–22] has been proposed theoretically by bridging the triphenyl-TM molecules with TM atoms, which show many exotic electronic properties, including topological insulating states [20, 21], and particularly, one such lattice made of triphenyl-Mn is shown to be a half metal [22].

In the present work, using first-principle calculations, we systematically studied the structural, magnetic, and electronic properties of the 2D free-standing HOMFs of triphenyl-TM lattices for all the 3d TMs from Sc to Cu. We found that the structures of this family of HOMFs have two types: buckled structure and twisted structure. Different TM elements lead to distinctively different electronic behavior, such as metallic vs. insulating and magnetic vs. nonmagnetic. Most interestingly, a few of them turn out to be half metals.

## Methods

Our first-principle calculations were based on the spin-polarized density functional theory (DFT) using the generalized gradient approximation (GGA) [23] in the form proposed by Perdew, Burke, and Ernzerhof (PBE) as implemented in the Vienna *ab initio* simulation package (VASP) code [24]. The projected augmented wave (PAW) method [25, 26] with a plane wave basis set was used. We applied periodic boundary conditions with 20-Å periodicity in the *z*-direction to avoid interaction between two 2D lattices in the neighboring unit cells. An energy cutoff of 400 eV and a 5 × 5 × 1 k-point mesh were adopted for optimization and total energy calculation. During structural relaxation, we set the convergence criteria for energy and force to be 10^-4^ eV and 0.01 eV/Å, respectively.

## Results and discussion

**Figure 1:**
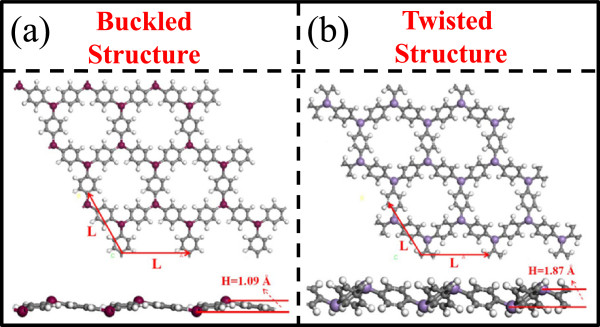
**Two kinds of structures of triphenyl-TM lattices: buckled structure and twisted structure. (a)** Top view and side view of triphenyl-Cr lattice as an example; **(b)** top view and side view of triphenyl-Mn lattice as an example. L is the lattice constant, H is the buckling height.

One unit cell of triphenyl-TM lattice contains two TM atoms connected by three benzene rings hexagonally. To obtain the lattice constants for the triphenyl-TM lattices, we optimize the lattice until in-plane stress components diminish. In Figure 1, we show the two kinds of structures of triphenyl-TM lattices: buckled structure and twisted structure. As an example, the triphenyl-Cr represents a typical buckled structure (Figure 1a) with the three benzene rings bonded to one Cr atom in C_3v_ symmetry, and the Cr atoms move out of the plane by a vertical distance of approximately 1.09 Å along the *z*-direction.

In contrast, the triphenyl-Mn lattice represents a typical twisted structure (Figure 1b) with three benzene rings bonded symmetrically to one Mn atom but each benzene ring twisted by an angle with respect to the axis along the two nearest Mn atoms, and the two Mn atoms are also buckled having a vertical distance of 1.87 Å along the *z*-direction. Table 1 summarizes the lattice constants and buckling heights and the C-TM bond length and C-TM-C bond angle of these 3d triphenyl-TM lattices. Interestingly, the lattices made of TMs from Sc to Cr, the left half row of 3d TMs, show buckled structures, while those made of TMs from Mn to Cu, the right half row of 3d TMs, show twisted structures. The Sc-to-Cr lattices have relatively larger lattice constants, C-TM bond lengths, and C-TM-C bond angles than the Mn-to-Cu lattices, consistent with the decreasing atom size from Sc to Ni. For Ni and Cu, in addition to the atomic-size effect, their d-orbitals strongly hybridize with the C sp-orbital, making their lattice constants increase; this effect can be seen from the electronic band structure calculation below.

**Table 1:** **Summary of the structure feathers of the triphenyl-TMs lattices**

	Element	***L***(Å)	***H***(Å)	***l***(Å)	*ϕ* (°)
Buckled structures	Sc	12.00	2.17	2.19	114.5
	Ti	11.80	1.64	2.07	113.1
	V	11.90	0.63	2.01	119.4
	Cr	11.80	1.09	2.01	116.7
Twisted structures	Mn	10.70	1.87	1.84	106.0
	Fe	10.20	2.58	1.82	101.0
	Co	10.15	2.58	1.81	101.1
	Ni	10.50	2.20	1.85	108.3
	Cu	11.00	1.88	1.93	114.6

*L* is lattice constant, *H* is the buckled height, *l* is the C-TM bond length in the lattice, and *ϕ* is the C-TM-C bond angle.

We also did molecular dynamics simulation [22] at room temperature to check the stability of such HOMF. For a free-standing triphenyl-TM lattice, the lattice is rather stable; only the benzene ring can rotate along the TM-benzene-TM axis, which may be impeded when the lattice is laid on a substrate.

**Figure 2:**
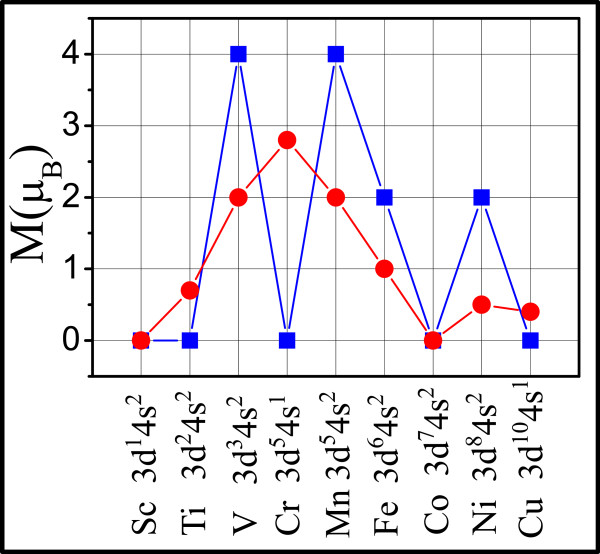
**Magnetic moments per unit cell (blue square) and per TM atom (red circle) for different triphenyl-TM lattices.**

Because these TMs contain unoccupied 3d orbitals, it is interesting to study their interatomic magnetic coupling in the HOMFs. Figure 2 shows the magnetic moments per unit cell and per TM atom for different HOMFs, along with the TM valence electron configurations (labeled on the *x*-axis). Most HOMFs are found magnetic except for the Sc and Co lattice. The V, Mn, and Fe lattices prefer ferromagnetic coupling; the magnetic coupling strengths (*E*_AFM_ - *E*_FM_) for V and Mn lattices are 0.371 and 0.295 eV, respectively, while the antiferromagnetic configuration of Fe is not stable. The Ti, Cr, and Cu lattices prefer antiferromagnetic coupling; the magnetic coupling strengths (E_FM_ - E_AFM_) are 0.073, 0.145, and 0.244 eV, respectively. For Ni, the energy difference between ferromagnetic and antiferromagnetic configuration is only 1 meV (*E*_FM_ lower).

The magnetic behavior of these HOMFs may be understood from simple electron counting. For Sc-Cr, both 4s and 3d electrons are involved in forming ‘covalent’ bond with three benzene rings. For Sc, all three valence electrons (two 4s and one 3d) are used to form bonds with three benzene rings, leaving no unpaired electron, so that Sc holds no magnetic moment. For Ti, V, and Cr, in addition to three valence electrons forming bonds, there are one, two, and three unpaired 3d electrons per TM atom, respectively, which give rise to 1, 2, and 3μ_B_ magnetic moments on each Ti, V, and Cr atom, respectively. This simple model agrees well with actual calculation results (Figure 2) which produce non-integer magnetic moments due to broadening of the atomic orbital into bands from TM-C bonding.

For Mn and Fe, the situation is different. Two 4s electrons are not involved in the bonding, only the 3d electrons bond to benzene rings. Due to local crystal field of C_3_ symmetry, the five 3d orbitals split into two groups: *d*_*xy*_, *d*_*yz*_, , and *d*_*zx*_ orbitals belong to E representation of C_3_ group, while  belongs to A representation. For triphenyl-Mn lattice, the *d*_*xy*_, *d*_*yz*_, , and *d*_*zx*_ orbitals of the Mn atom have lower energy, leading to parallel spin alignment of two extra 3d electrons that are not involved in bonding, giving rise to 2μ_B_ on each Mn atom and 4μ_B_ per unit cell. For triphenyl-Fe lattice, the  orbital of Fe atom has lower energy, two of the three un-bonded 3d electrons will occupy the two antiparallel  x orbital, leaving behind one unpaired 3d electron occupying the higher level, leading to 1μ_B_ on each Fe atom and 2μ_B_ per unit cell.

**Figure 3:**
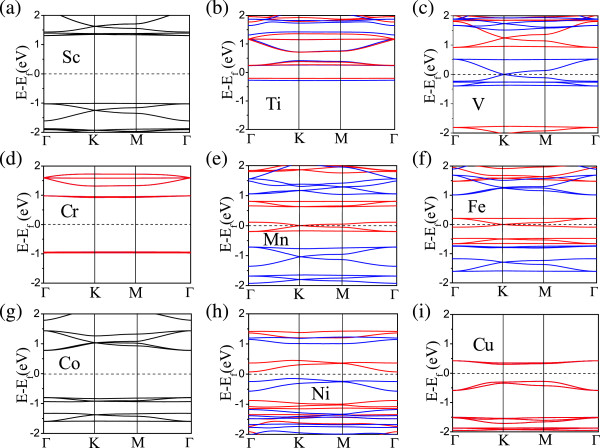
**Spin-polarized band structures of the triphenyl-TM lattices. (a-i)** Spin-polarized band structures of the triphenyl-TM lattices from Sc to Cu. Black curves in **(a)** and **(g)** mean nonmagnetic bands; blue and red curves in other plots are the spin-up and spin-down bands, respectively. Only spin-down (red) bands are shown in **(d)** and **(i)** for AFM lattices.

For Co, Ni, and Cu, the hybridization between TM and C orbitals becomes too strong. Consequently, we cannot interpret the magnetic behavior based on the above simple argument. Specifically, for the Co lattice, our calculation indicates that the strong hybridization makes the Co 3d orbit become fully filled without magnetism and the Ni magnetic moment be a non-integer number along with non-integer moments on C atoms. The differences in the involvement of 4 s orbitals in bonding are probably the reason why the Sc-to-Cr lattices with s-orbital bonding show buckled structure and the others without s-orbital bonding show twisted structures.To further reveal the electronic properties of these HOMFs, we show their spin-polarized band structures in Figure 3. For Sc and Co, they are nonmagnetic, with degenerate spin-up and spin-down bands. They are insulators with a DFT band gap more than 1.5 eV. For V, Mn, and Fe, the spin-up (blue) and spin-down (red) bands split away from each other, resulting in ferromagnetic half metals. The Ti, Cr, and Cu lattices are antiferromagnetic, with degenerate spin-up and spin-down bands and opposite spins on the two TM atoms in the unit cell. The Ni lattice is a magnetic semiconductor.

An interesting property of these half-metallic HOMFs(V, Mn, and Fe) is that they have both Dirac bands, with Dirac points at K and K′, and flat band, due to the underlying hexagonal lattice symmetry [21, 22]. We note that inclusion of spin-orbital coupling will open small band gaps but without affecting magnetic properties, as shown before for Mn lattice [21, 22], and similar behavior is found here for Fe and Cr lattice. Depending on the location of the Fermi level, quantum anomalous Hall effect may be realized for these systems at low temperatures [21, 22].

**Figure 4:**
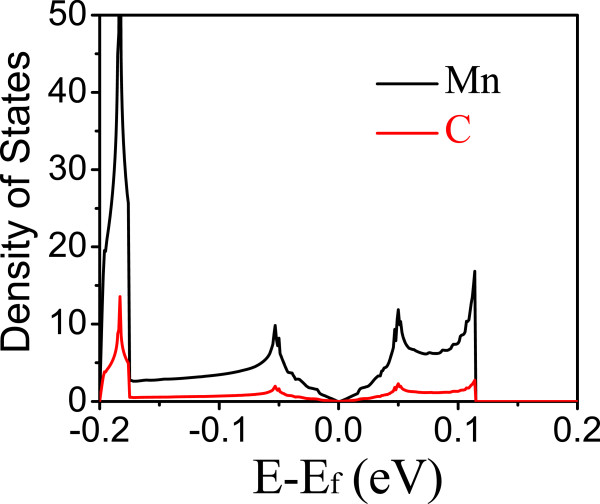
**Partial density of states near the Fermi level for triphenyl-Mn lattice.**

To further understand where the states near the Fermi level come from, we calculated partial density of states near the Fermi level for those half-metallic HOMFs, as shown in Figure 4 for Mn and C atoms in the triphenyl-Mn lattice. Only the spin-down band exists near the Fermi level. We can see that they are mainly from Mn d-states, in agreement with what we discussed above, which are degenerate *d*_*xy*_, *d*_*yx*_, , and *d*_*zx*_ states, while  state is approximately 0.7 eV above the Fermi level (see Figure 3e). Electronic hopping among these d-states in a hexagonal lattice, ‘mediated’ through the benzene rings in between them, forms a Dirac state at the Fermi level. The triphenyl-Fe lattice behaves very similarly, except that the  states are occupied, about 0.6 eV below the Fermi level (see Figure 3f). The triphenyl-V lattice is also similar, as shown in Figure 3c.

**Figure 5:**
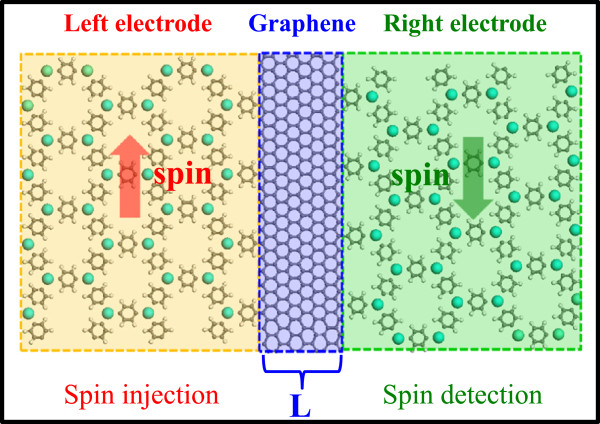
**Schematic illustration of a HOMF-graphene-HOMF spintronic device.**

The DFT band gaps for the insulating spin channel of these half-metallic 2D HOMFs are more than 1.5 eV, making them ideal spin-injection or spin-detection materials. Another important component of a spintronic device is a spin conductor (or carrier) which transports spin as fast and as long as possible. Graphene has been proposed as an ideal 2D spin conductor because of its small SOC and long spin-coherence length [27, 28]. Considering both the 2D nature of HOMF and graphene, and high bonding compatibility of molecular ligands in HOMF with graphene, we propose an attractive design of spintronic devices built from two HOMF-graphene-HOMF heterojunctions as shown in Figure 5. On the left side, half-metallic HOMF is used as spin injector and one the right as spin detector; while the graphene works as the spin conductor in the middle. The transport properties, as well as functionality of such devices, will be interesting topics for future studies.

## Conclusions

In summary, we systematically studied the structural, magnetic, and electronic properties of a kind of HOMF containing 3d transition metals, using first-principle calculations. We found that their structures can be divided into two groups: buckled versus twisted, probably due to the degree of 4s-3d orbital hybridization involved in forming bonds with benzene rings. Some of these HOMFs (V, Mn, and Fe) favor ferromagnetic coupling and show half metallicity with the size of the band gap in the insulating spin channel larger than 1.5 eV, which makes these HOMF half metals potential 2D spin-injection or spin-detection materials. Combining with a good spin-conducting material, such as graphene, we also propose a possible design of 2D spintronic device.
